# Editorial: Navigating sustainability: addressing esports winter challenges

**DOI:** 10.3389/fspor.2025.1766689

**Published:** 2026-01-12

**Authors:** Jeffrey Levine, Csilla Csukonyi, Michael Wagner, Dávid Papp

**Affiliations:** 1 Drexel University, Philadelphia, PA, United States; 2 University of Debrecen, Debrecen, Hungary

**Keywords:** esports, governance, professionalization, sustainability, wellbeing

## Introduction

Over the past few years, the phrase “esports winter” has come to describe a period of correction and consolidation following a decade of rapid expansion in competitive gaming. Investor pullbacks, layoffs, restructurings, and reduced valuations have prompted concerns about the industry's trajectory. Rather than using this moment to question esports' viability, it is more productive to treat it as a stress test that reveals which parts of the ecosystem are resilient and which remain fragile. This shifts attention from whether esports will continue to what kinds of futures are actually sustainable.

This special issue explores these questions through the lens of sustainability. Esports winter is not simply an economic story; it is also about legal structures, business models, labor and health conditions, and the organizational capacities that support competitive play. This collection, “*Navigating Sustainability: Addressing Esports Winter Challenges*”, brings together five studies that examine sustainability from complementary angles: industry structure, sponsorship strategies, player health, psychological resilience, and grassroots club-based esports. These contributions collectively assess the field's underlying structures and use esports winter as an opportunity to reconsider what sustainable practice might look like in the years ahead.

The Research Topic includes five original research articles that span legal doctrinal and economic analysis, portfolio mapping, cross-sectional survey work, and qualitative interviews. Novák et al. examine the legal and economic foundations of the industry. Zhu et al. analyze sponsorship portfolios in the League of Legends Pro League (LPL). Kurniawan et al. document health risks among mobile esports players in Jakarta. Papp et al. focus on psychological characteristics that underpin performance. Baumann et al. explore grassroots esports initiatives in Norwegian sports clubs. These studies, cumulatively, underscore sustainability challenges across multiple levels of the esports ecosystem.

Novák et al. address esports winter at its structural core by asking why competitive gaming has struggled to become an independent industry. They show how the legal primacy of game publishers, grounded in intellectual property rights and international treaties, gives publishers full control over competition formats, entry, and monetization. All major esports titles remain under publisher control, and esports functions more as a supplemental marketing tool than a standalone profit engine. Esports winter is therefore interpreted as a market correction following speculative investment and inflated comparisons with traditional sport. Within this structure, stakeholders gain influence less through formal autonomy than through legal literacy, strategic partnerships with publishers, and professional management.

Zhu et al. shift attention from law to revenue by mapping sponsorship portfolios in the LPL. Using data on club and league sponsors, they quantify both continuity and diversification across industry categories. Tencent, as the league operator, exhibits a mature continuity strategy while maintaining a diversified sponsor base, securing more predictable cash flows and preserving the premium value of sponsorship slots. Many clubs, by contrast, display fragmented and short-lived portfolios, and only a minority combine continuity with diversification. What distinguishes these approaches is less competitive success than differences in managerial capacity and strategic orientation. In a volatile environment, clubs with stable and diversified sponsorships gain financial resilience and brand differentiation, while those lacking such capacities remain more exposed.

Kurniawan et al. bring the discussion to the level of well-being by examining health risks among professional and casual mobile esports players in Jakarta. In a sample of 94 athletes, roughly two-thirds report musculoskeletal complaints, with the neck, shoulder, hand, and wrist most commonly affected. All participants indicate experiencing at least one eye problem, with eye fatigue especially prevalent. The majority of players report low levels of physical activity and sleep between six and seven hours per night, even though body mass index values are generally normal. The pattern that emerges points to posture, prolonged static play, repetitive movements, and limited recovery as the primary sources of risk. Sustainable practice will require greater attention to ergonomics, structured breaks, physical conditioning, and eye health.

Papp et al. examine psychological factors that encourage esports participation by identifying mental characteristics that influence performance among e-athletes. Through focus groups and interviews, they identified 17 relevant traits, with resilience emerging as the most frequently mentioned and central characteristic for becoming and remaining a professional player. These traits cluster into six broader domains: Developmental Motivation, Attentional Skills, Game Intelligence, Perseverance and Stress Management, Motor Skills, and Social Skills. Rather than treating mental skill as a narrow performance variable, the framework repositions high-level play as dependent not only on technical and motor abilities, but also on coping with pressure, sustaining focus, creatively and critically applying game-specific knowledge, and functioning effectively within a team. The clusters map where talent development and psychological training may be most effective, pointing toward developmental pathways that foster more sustainable, resilient careers in esports.

Baumann et al. conclude the collection at the grassroots level by examining how volunteer-led esports programs are sustained within Norwegian sports clubs. Interviews with 15 volunteer leaders emphasize the central role of local community impact, with programs frequently described as safe and inclusive spaces for youth who feel disconnected from traditional sport. However, these initiatives often operate with limited institutional support and rely heavily on a small number of individuals. This creates ongoing challenges related to recruitment and resource management. Gaps in esports-specific expertise further constrain program development. Long-term sustainability at the grassroots level depends on stronger support structures, targeted training, and clearer recognition of esports within club sport systems.

These studies, when considered holistically, illustrate that esports winter is best understood not as a setback but as a moment of recalibration. They demonstrate that sustainability in esports is shaped by conditions operating at multiple levels of the ecosystem, rather than within any single domain. Addressing sustainability in esports will require coordinated attention across these interconnected dimensions rather than isolated reforms. Doing this work will help clarify what a sustainable future for esports requires and how the field can reinforce the foundations needed to support it.

